# Effect of Acidification on the Properties of Films Developed from Carboxymethylcellulose and Jabuticaba Anthocyanin Extract (*Plinia Cauliflora*)

**DOI:** 10.1021/acsomega.5c11756

**Published:** 2026-03-18

**Authors:** Carolina da Silva Ponciano, Cristiane Patrícia de Oliveira

**Affiliations:** 67658 Southwest Bahia State University, Itapetinga 45031-900, Brazil

## Abstract

This study evaluates acidification as a formulation strategy in carboxymethylcellulose (CMC)-based films incorporating anthocyanins extracted from jabuticaba peel (*Plinia cauliflora*), aiming to develop intelligent and biodegradable materials for food packaging. To the best of our knowledge, anthocyanins derived from jabuticaba peel have not previously been incorporated into CMC-based film systems, particularly with a systematic assessment of formulation acidification. Three film formulations were prepared: a control film (FC, pH 6), a nonacidified film containing anthocyanins (FSA, pH 5), and an acidified film containing anthocyanins (FCA, pH 3), allowing the effects of anthocyanin incorporation and matrix acidification to be evaluated independently. Spectrophotometric analysis confirmed the pH-responsive behavior of the anthocyanins, with acidification promoting a deeper red hue and improved chromatic stability. Anthocyanin incorporation increased film thickness and reduced tensile strength and elastic modulus, while Fourier-transform infrared spectroscopy indicated interactions between anthocyanins and the CMC matrix. Importantly, thermal stability was maintained, and acidification significantly enhanced color intensity without compromising thermal properties. Overall, these findings support the potential application of the developed films in solid or semisolid food packaging systems.

## Graphical abstract

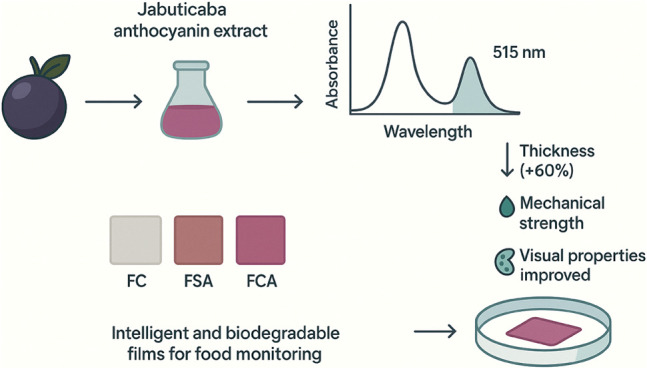

## Introduction

Intelligent packaging systems are functional materials that monitor and provide real-time information on product quality during transport, storage, and distribution.^1^ Among the main examples reported in the literature are indicator sachets and films designed to detect changes in pH, microbial growth, temperature fluctuations, and freshness loss.^2,3^ Such technologies are especially relevant to the food industry, as they enable immediate visual communication of a food product, usually through observable color changes. The development of intelligent films contributes to reducing food waste, increasing consumer safety, and promoting sustainable practices within the packaging industry.^4^

Natural pigments such as anthocyanins have emerged as particularly promising materials for these packaging formulations. Although the number of anthocyanin-based pH-responsive films remains limited, existing studies demonstrate their potential due to the natural origin, nontoxicity, and pronounced chromatic response of these compounds to environmental changes. Anthocyanins extracted from fruits and agro-industrial residues have attracted growing attention, particularly in the context of sustainable and biodegradable materials.^5–8,10,11^

Anthocyanins are water-soluble flavonoid compounds responsible for the coloration of various flowers, fruits, and vegetables. Their chemical structure confers high pH sensitivity, enabling them to act as visual indicators across a wide pH range. Under strongly acidic conditions (pH < 3), anthocyanins predominantly exist as the flavylium cation, exhibiting red coloration. At mildly acidic to neutral pH, they may undergo structural transformations into quinoidal bases, carbinol pseudobase (colorless), and chalcone-related forms, which may exhibit yellowish tones. Under alkaline conditions, degradation reactions prevail, resulting in color loss.^3,12,13^ The stability and chromatic behavior of anthocyanins are influenced by pH, temperature, exposure time, and molecular substitution patterns. Lower pH values favor the protonated forms and enhance color stability, whereas increasing pH promotes structural rearrangements that compromise chromatic intensity.^14–17^

The incorporation of anthocyanins into natural polymer matrices has received increasing attention, particularly for the potential development of biodegradable, environmentally sustainable materials.^18,19^ This trend is driven by the need to replace petroleum-derived synthetic packaging, which is mostly nonbiodegradable and nonrenewable.^20^ Recent studies include Huynh et al.,^21^ who incorporated red onion peel extracts into alginate emulsions with and without CMC, thereby maintaining antioxidant and antimicrobial activity in strawberries. Alnadari et al.^22^ developed CMC-gum arabic (GA) films with anthocyanins from *Cinnamomum camphora* peel residues, improving mechanical, physical, and bioactive properties while providing pH and ammonia sensitivity for food freshness monitoring.

Among natural polymers used in film production, CMC, a cellulose-derived polysaccharide, stands out due to its high-water solubility, tastelessness, odorlessness, nontoxicity, and excellent capacity to form stable matrices. It facilitates the incorporation of bioactive compounds, such as natural pigments, and provides a barrier against environmental factors, contributing to food protection and extending shelf life.^23–28^ Upon incorporation of bioactive compounds, CMC-based films generally exhibit increased thickness, modified mechanical properties, altered color, and enhanced antioxidant or antimicrobial activity, depending on the type and concentration of the bioactive substance, as demonstrated by Huynh et al.^21^ and Alnadari et al.^22^

Despite advances in anthocyanin-based intelligent films, pigment stability in polymeric matrices remains challenging, and studies specifically addressing matrix acidification as an independent formulation strategy are still limited. In addition, monopolymeric CMC films incorporating anthocyanins extracted from jabuticaba peel (*Plinia cauliflora*) have not yet been reported, despite the high anthocyanin content and agro-industrial relevance of this byproduct. Therefore, this study aims to evaluate acidification as an independent formulation parameter in films prepared exclusively from CMC containing jabuticaba peel anthocyanins, allowing the individual effects of anthocyanin incorporation and controlled matrix acidification on chromatic response, physicomechanical properties, and thermal stability to be systematically assessed.

Thus, this work contributes to advancing sustainable intelligent packaging research by offering biodegradable alternatives with the potential to replace conventional synthetic materials, while adding functional value and reducing environmental impacts.

## Materials and Methods

### Extraction and Determination of Total Anthocyanin Concentration in the Extract

Jabuticaba fruits (Plinia cauliflora) were obtained from Fazenda Evandro, located in Itapetinga, Bahia, Brazil. After visual selection and discarding fruits with apparent imperfections, the fruits were washed and frozen at −18 °C until use. Anthocyanin extraction was performed in triplicate, based on methodologies described by Tena and Asuero^29^ and Lima et al.^30^

The fruits were thawed, manually pulped, and the peels were then ground using an industrial blender (KD Eletro, 800 W, 1 HP, 3850 rpm). The ground samples were weighed on an analytical balance (Shimadzu, Japan, model AY2200) and immersed in acidified water with 1.5 N HCl (pH adjusted to 2) at a ratio of 1 part peel to 4 parts solvent (w/v). Samples were manually agitated for 5 min and then left to rest for 24 h at room temperature (25 ± 2 °C), protected from light. After this period, the extract was filtered through qualitative filter paper (Unifil, Germany; thickness 16 μm, filtration rate 20–25 s).

The total anthocyanin concentration was determined by UV–vis spectrophotometry following the Beer–Lambert law, according to the methodology described by Lee et al.^31^ An aliquot of 3 mL of the extract was transferred to a quartz cuvette (1 cm path length), and absorbance was measured at 535 nm using a UV-1800 spectrophotometer (Shimadzu, Japan). Readings were performed in triplicate, and the mean absorbance values were used to calculate the molar concentration (mol·L^–1^) according to eq 1. In the equation, ε represents the molar absorptivity, adopting the average value of 98.2 L·mol^–1^ ·cm^–1^, as reported by Francis^32^ for anthocyanins in acidic media.

1 Abs=ε.l.c

### Film Preparation

Films were produced by the casting method, with adaptations from Halász and Csóka^33^ and Pereira et al.^34^ The adaptation consisted of replacing the polymer matrices originally based on chitosan and poly­(vinyl alcohol) with carboxymethylcellulose, while maintaining the same film-forming principle and casting procedure. Control films (FC, pH 6) were prepared from a 1% (w/v) CMC dispersion containing 17% (w/w) glycerol as a plasticizer, based on CMC weight.

For films containing anthocyanins, the film-forming dispersion was prepared using one part extract (0.0023 M) to three parts solvent (w/v). The extract concentration was selected based on preliminary trials and visual screening, aiming to ensure adequate color intensity and pH responsiveness while maintaining film homogeneity and mechanical integrity. The formulations were divided into two treatments: films containing anthocyanins without pH adjustment (FSA, pH 5) and films containing anthocyanins with pH adjustment (FCA, pH 3), pH adjustment was performed by adding 1.5 N HCl solution. The choice of pH 3 and 5 was based on previous reports of anthocyanin stability under acidic conditions,^14,15^ where protonated forms exhibit higher color intensity and stability.

This experimental design allows the independent evaluation of the effects of anthocyanin incorporation and matrix acidification on the physicochemical and functional properties of the films. Film-forming dispersions were poured onto 33 × 23 cm rectangular glass plates and dried in an air-circulated oven at 50 ± 2 °C for 6 h. After drying, films were carefully removed from and stored in a desiccator with silica gel, protected from light, until analysis. All formulations were prepared in triplicate.

## Characterization of Films

### Color Analysis

The extracts and films were evaluated under different pH conditions using specific buffer solutions: 0.1 mol·L^–1^ sodium citrate buffer (trisodium citrate + anhydrous citric acid), adjusted to pH 3, 4, and 5; 0.1 mol·L^–1^ sodium phosphate buffer (monosodium phosphate + disodium phosphate), adjusted to pH 6, 7, and 8; and 0.05 mol·L^–1^ sodium carbonate buffer (sodium carbonate + sodium bicarbonate +2 mol·L^–1^ NaOH), adjusted to pH 9 and 10.

Color coordinates were determined by digital image analysis using the Color Grab app, following the methodology described by Shahvalinia.^23^ For the extracts, 2 mL of sample and 1 mL of buffer were placed in Petri dishes and allowed to rest for 5 min prior to image capture. For the films, 5 mL of buffer solution was added to the predried samples, promoting solubilization under the same conditions used for the extracts.

Photographs were taken with a Xiaomi Redmi Note 8 smartphone (48 MP) under standardized conditions: a closed environment with a white background and a fixed distance of 27.5 cm. The images were analyzed to determine the colorimetric coordinates of lightness (L*), red-green (a*), and yellow-blue (b*).

In addition to the L*, a*, and b* coordinates, the total color difference (ΔE*) was calculated for the films according to eq 2, as well as the whiteness index (WI), determined according to eq 3 The variables ΔL*, Δa*, and Δb* represent the differences between the color coordinates of the anthocyanin-containing films and those of the control film (*L*_0_*, *a*_0_*, *b*_0_*), respectively.

2 $$\Delta E^{*}=\surd {\left(\Delta L^{*}\right)}^{2}+{\left(\Delta a^{*}\right)}^{2}+{\left(\Delta b^{*}\right)}^{2}$$

3 $$\mathrm{WI}=100-\surd {\left(100-L^{*}\right)}^{2}+{\left(a^{*}\right)}^{2}+{\left(b^{*}\right)}^{2}$$

### UV/VIS Spectroscopy

Optical absorption measurements of the films and extract in the visible region (Vis) were performed using a spectrophotometer (UV-1800, Shimadzu, Japan), scanning the wavelength range from 400 to 800 nm, according to the methodology described by Hosseini et al.,^35^ with the spectral range restricted to the visible region in order to specifically assess the chromatic behavior of anthocyanins. For the extract analysis, samples were previously mixed with buffer solutions adjusted to different pH values (3–10) at a ratio of 2 mL extract to 1 mL buffer solution. After 5 min of contact, the samples were transferred to glass cuvettes with a 1 cm optical path length and subjected to spectral scanning. For the films, samples measuring 3 × 1 cm^2^ were immersed in 5 mL of buffer solution and kept in contact for 5 min. Then, a 3 mL aliquot of the solution was transferred to glass cuvettes, and spectral scanning was performed under the same conditions as for the extracts.

### Film Thickness

Film thickness was determined according to the methodology described by Escobar et al.,^36^ using a digital micrometer (PIK B-Pantec, Model IP54, São Paulo, Brazil) with a resolution of 0.001 mm. Samples were positioned horizontally, and measurements were taken at five random points across the surface of each film. Results were expressed in millimeters (mm) as the arithmetic means of the readings obtained.

### Transparency Percentage

The transparency test was conducted following the methodology described by Pérez-Córdoba et al.^37^ Films were cut into approximately 3 × 1 cm^2^ pieces and placed in the sample compartment of the spectrophotometer. The amount of electromagnetic radiation transmitted through the sample was recorded and compared to the air transmittance (blank), used for instrument calibration. Measurements were taken at a wavelength of 670 nm. Transparency was quantified using eq 4, where T% = Percentage of transparency, *I* = Transmittance (%), and δ = Thickness (mm).

4 $$T\%670=\frac{\log I}{\delta }$$

### Mechanical Properties

The mechanical properties of the films were determined according to ASTM D882–18,^38^ with minor adaptations regarding specimen size and testing speed to suit the CMC-based films used in this study. Tests were conducted using a Brookfield CT3 texture analyzer (Brookfield Engineering, USA) with a load capacity of up to 25 kg. Specimens measuring 2.7 × 10.8 cm^2^ were vertically fixed in the equipment, maintaining an initial grip distance of 10 cm. Tensile force was applied vertically at a constant speed of 1.5 mm·s^–1^ until material rupture. For each formulation, three independent films were evaluated, with seven specimens analyzed per film.

From the obtained data, the following parameters were calculated: Elongation (%), Maximum Tensile Strength (MPa), and Young’s Modulus (MPa) using eqs 5, 6, and 7, respectively. Where ε = Elongation (%), At = Maximum deformation (m), DG = Initial grip distance (m), φ= Maximum stress, F = Maximum force (N), A = Cross-sectional area (m^2^), MY = Young’s Modulus, Δφ = Stress variation (MPa), and Δε = Elongation variation within the elastic deformation region (m).

5 $$\varepsilon =\frac{\mathrm{At}}{\mathrm{DG}}\times 100$$

6 $$\varphi =\frac{F\max}{A}$$

7 $$MY=\frac{\Delta \varphi }{\Delta \varepsilon }$$

### Fourier Transform Infrared Spectroscopy (FTIR)

Film samples measuring 3 × 1 cm^2^ were analyzed using a Fourier Transform Infrared Spectrometer (Cary 630, Agilent Technologies Inc., Santa Clara, USA), coupled with an Attenuated Total Reflectance (ATR) accessory equipped with a diamond crystal cell and a deuterated triglycine sulfate (DTGS) detector. The diamond crystal has a sampling area of approximately 1 mm in diameter and an active area of 200 μm, allowing infrared radiation penetration of about 2 μm at 1700 cm^–1^.

Spectra were collected in absorbance mode with a resolution of 4 cm^–1^ over the mid-infrared region, covering the range from 4000 to 600 cm^–1^. Sixty-four scans were performed per sample, with a total acquisition time of approximately 30 s, at a controlled temperature of 25 ± 2 °C. A background spectrum was recorded before each sample measurement. Data were processed using Microlab Resolution Pro software (Agilent, Santa Clara, USA).

### Water Vapor Permeability

Water vapor permeability (WVP) was determined following the ASTM E96–00 ^9^ method. Circular films, approximately 3 cm in diameter, were placed over plastic permeation cups containing dry silica gel (105 °C/1h) and sealed with plastic lids. The cups were initially weighed and then placed in a desiccator containing distilled water (relative humidity = 100%; vapor pressure = 32.23 mmHg), maintained in a climate-controlled environment (20 °C ± 2 °C).

The cups were weighed every 24 h until a constant weight was achieved. Analyses were performed in duplicate for each film, following the general replication scheme described in the Statistical Analysis section. Water vapor permeability was calculated using eq 8, where WVP = water vapor permeability (g·m^–1^·s^–1^·mmHg^–1^); G = mass gain (g); δ = film thickness (m); A = film area (m^2^); T = exposure time (s); P1–P2 = water vapor pressure gradient (mmHg).

8 $$\mathrm{WVP}=\frac{G\times \delta }{A\times T\left(P1-P2\right)}$$

### Solubility

The solubility test was conducted following the methodology described by Pérez-Córdoba et al.^37^ Films were cut into squares measuring 20 mm on each side and conditioned in a desiccator containing dry silica gel for 24 h. Subsequently, residual moisture was removed by drying in an oven at 105 ± 2 °C for 15 h. The weight of the dried samples was recorded as the initial mass (Mi) for solubility analysis.

The dried samples were immersed in Erlenmeyer flasks containing 50 mL of distilled water and kept under agitation on an orbital shaker (Model MA-140/CF, Brazil) at 25 ± 2 °C and 60 rpm for 24 h. After this period, the solution was filtered using qualitative filter paper (Unifil, Germany; thickness 16 μm, filtration rate 20–25 s).

The remaining residue was dried again as previously described, and the weight after the second drying was recorded as the final mass (Mf). Solubility was calculated using eq 9, where S (%) = solubility percentage, Mi = initial mass (after first drying) (g), Mf = final mass (after second drying) (g).

9 $$S\left(\%\right)=\frac{Mi-Mf}{Mi}\times 100$$

### Thermogravimetry (TGA) and Differential Thermal Analysis (DTA)

Thermogravimetric analysis (TGA) and differential thermal analysis (DTA) were performed using approximately 10 mg of sample, submitted to a TGA/DTA calorimeter Test Instrument LINSEIS (model STA PT 1000 Simultaneous, Germany). The samples were subjected to a heating ramp ranging from 25 to 900 °C in an oxygen atmosphere with a heating rate of 10 °C/min. The results obtained were analyzed to determine the samples thermal transitions and decomposition behaviors.

### Film Stability

Film samples, with approximate dimensions of 3 × 1 cm^2^, were stored in sealed Petri dishes, kept away from light and at room temperature (25 ± 2 °C), to analyze the colorimetric stability of anthocyanin incorporated into the carboxymethylcellulose polymer matrix over time.

The samples were photographed weekly for 8 weeks, using a Xiaomi Redmi Note 8 smartphone (48 MP) under standardized conditions: inside a closed box with a white background at 27.5 cm. The images obtained were analyzed to determine the colorimetric coordinates L*, a*, b*, and to calculate ΔE* using the Color Grab application.

### Application of Films

The practical application of the films was simulated using pasteurized milk to evaluate the colorimetric response to pH variations during storage. Film samples (3 × 3 cm^2^) were placed in Petri dishes containing 15 mL of pasteurized milk and kept at room temperature (25 ± 2 °C), protected from light, for up to 48 h. Images of the films were captured at 0, 24, and 48 h and analyzed to determine the colorimetric coordinates L*, a*, and b*, according to the conditions described in the previous section. Samples containing only milk were used as controls.

### Statistical Analysis

The experiment was conducted using a completely randomized design (CRD). All film formulations were prepared in triplicate, and each characterization analysis was performed using at least three independent samples per formulation, unless otherwise specified. The obtained data were subjected to analysis of variance (ANOVA), followed by Tukey’s multiple comparison test, with a significance level of *p* < 0.05. Analyses were performed using SAS OnDemand for Academics software (SAS Institute Inc., Cary, NC, USA), University Edition. This procedure allowed the evaluation of the effect of anthocyanin extract incorporation on the properties of the films.

## Results and Discussion

### Evaluation of Extracts

Figure 1 and Table 1 present the images and values of the L*, a*, b* color coordinates of the jabuticaba peel extract at a concentration of 0.0023 M, subjected to different pH values. Visual inspection of the images reveals distinct colors of the extract under varying pH conditions. This color difference is confirmed by the a* and b* coordinate values, which indicate that at pH 3 and 4, the extract exhibits pinkish hues, while at pH 5 and 6 it becomes colorless, and from pH 7 to 10 it shows earthy yellow tones. The a* coordinate was primarily responsible for the hue changes in the extracts. Regarding L*, the extracts maintained high luminosity across all pH ranges, indicating stability in this parameter.

**Figure 1:**
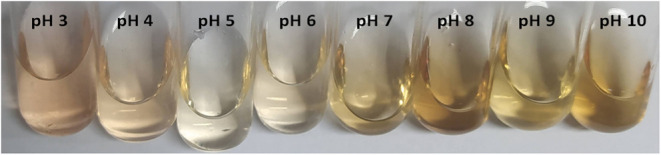
Anthocyanin extract from jabuticaba peels subjected to different pH values.

**Table 1:**
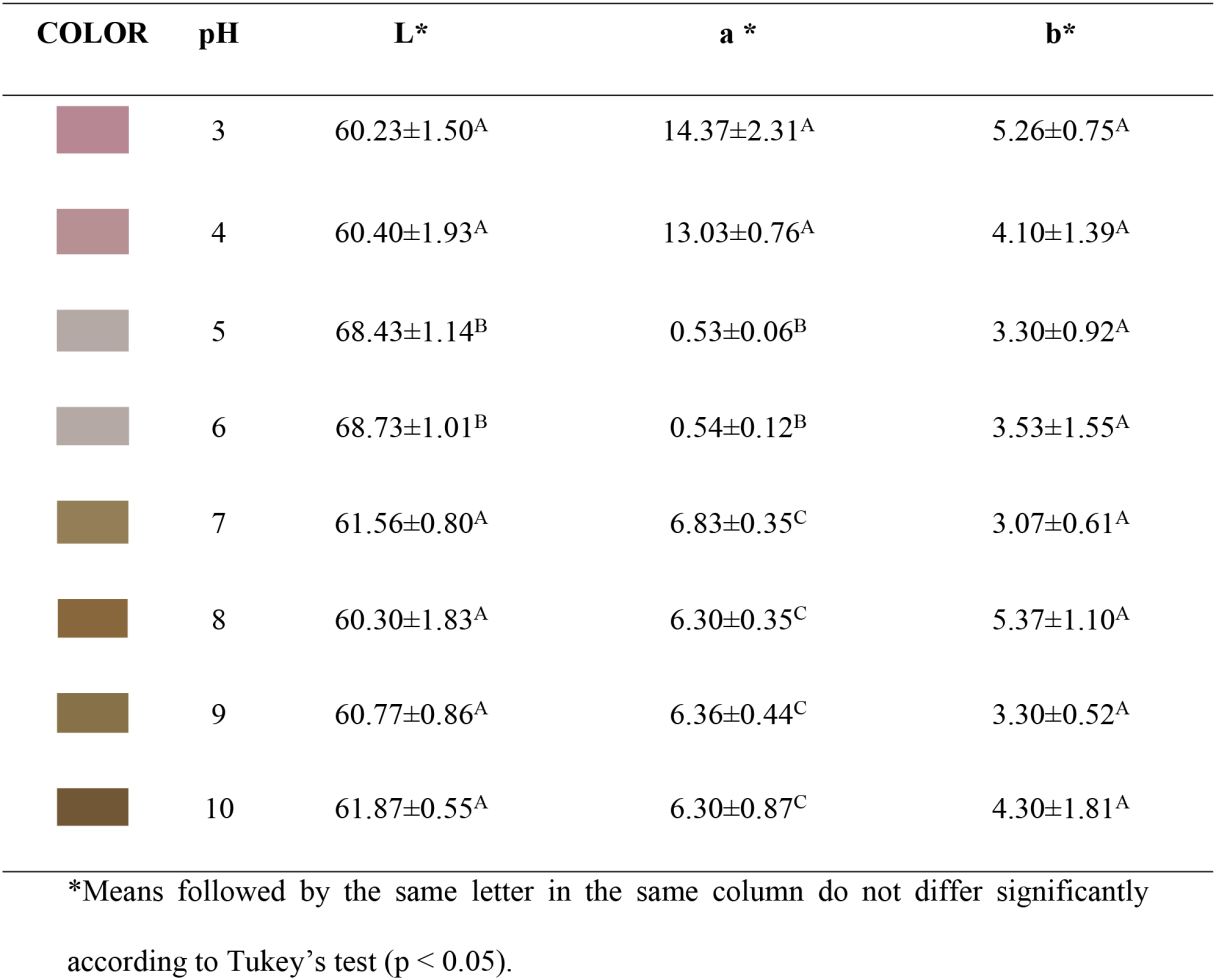
Variation of Color Coordinates of Anthocyanin Extracts as a Function of pH Changes in the Aqueous Medium

This color variability is associated with chemical structural modifications of the anthocyanins present in the extract. According to Zhang,^11^ under acidic conditions (pH 3 and 4), the flavylium cation form predominates, imparting pink hue to the extract. As the pH approaches 5 and 6, the pseudobase carbinol predominates, resulting in a colorless extract. At pH 7 to 10, chalcone formation occurs, responsible for the yellowish tones, a phenomenon also observed in the present study. These observations highlight the sensitivity of anthocyanins to pH; a principle applied later in film acidification strategies (FCA vs FSA).

The absorption spectra of the anthocyanin extract from jabuticaba peel, in the range of 400 to 700 nm and at pH values from 3 to 10, are presented in Figure 2. At pH 3, the extract exhibited an absorption peak at 515 nm, which is characteristic of the flavylium cation form of anthocyanins. As the pH increased to moderately alkaline values (4–6), the peak shifted to 520 nm with a gradual decrease in intensity, indicating structural changes in the anthocyanins. From pH 7 onward, the intensity of the absorption peak at 520 nm decreased substantially, reflecting a significant reduction in the protonated form of anthocyanins and, consequently, in color intensity.

**Figure 2:**
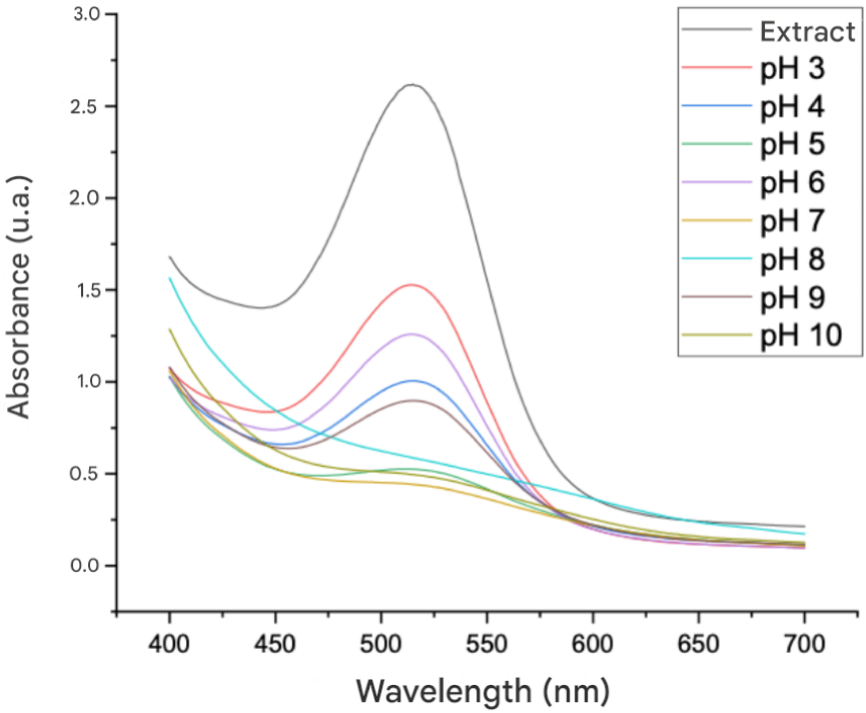
Absorption spectra in the UV/Visible regions (400–700 nm) of anthocyanin extract from jabuticaba peel at different pH values (3.0–10.0).

According to Căta et al.,^39^ anthocyanins exhibit strong absorption in the visible region (490–550 nm) due to the presence of conjugated double bonds, which are highly sensitive to pH changes. Increasing the pH leads to deprotonation of the flavylium cation and the formation of species such as the carbinol pseudobase, which has lower conjugation of double bonds. This structural modification results in a hypochromic shift (reduced absorption intensity) and spectral shift. At higher pH values, the predominance of structurally altered forms explains the drastic reduction in the color intensity observed in the extracts.

The pH variation directly influenced the optical properties of the extract, reflecting significant structural changes in the anthocyanin molecules. The combined analysis of color and absorption spectra enabled a better understanding of the extract’s behavior in response to pH changes, highlighting its potential for applications in pH-sensitive systems, especially in intelligent biodegradable films that function as visual indicators of pH variation.

## Film Characterization

### Thickness and Mechanical Properties

The data for thickness, tensile strength, elongation, and Young’s modulus of the FC, FCA, and FSA films are presented in Table 2. The thickness of FCA and FSA films did not differ significantly from each other; however, when compared to FC, the incorporation of anthocyanin extract led to a considerable increase in film thickness. This increase can be attributed to the higher solids content in the casting solution, as higher concentrations of solids provide more material for film formation after solvent evaporation, resulting in thicker films.

**Table 2:** Thickness Measurements and Mechanical Properties of FC, FCA, and FSA Films^a^

Films	Thickness (mm)	Maximum stress (MPa)	Stretching (%)	Modulus of elasticity (MPa)
FC	0.05 ± 0.002^A^	2.37 ± 0.55^A^	7.01 ± 1.34^A^	34.57 ± 10.03^A^
FCA	0.08±0.01^B^	0.69 ± 0.03^B^	11.38 ± 1.86^AB^	6.14 ± 0.87^B^
FSA	0.07±0.01^B^	1.21 ± 0.55^B^	18.72 ± 5.14^B^	7.06 ± 3.93^B^

a Mean values followed by the same letter in the same column do not differ from each other, according to the Tukey test (*P* < 0.05).

Therefore, the presence of anthocyanins was the determining factor for the increase in film thickness. Similar results were reported by Yong et al.^40^ and Prietto et al.,^41^ who also observed an increase in the thickness of films with anthocyanin incorporation, emphasizing that thickness is directly related to the solids content in the formulation.

Film thickness is a critical parameter, as it allows for the assessment of the manufacturing process homogeneity, the reproducibility of results, and facilitates comparisons between the properties of different films. Moreover, this parameter directly influences properties such as water vapor permeability, light transmittance, and mechanical behavior, including tensile strength.^42^

The control film (FC) exhibited higher tensile strength, lower elongation, and greater rigidity compared to the films modified with anthocyanins. The FSA and FCA films showed partially similar mechanical properties, although the FCA film exhibited lower tensile strength and elongation than the FSA. This difference is attributed to the acidification in FCA, which enhances hydrogen bonding between anthocyanins and the CMC matrix, restricting chain mobility compared to the nonacidified FSA film.

The incorporation of anthocyanin extract likely acted as a plasticizer, reducing intermolecular interactions between polymer chains through hydrogen bonding between hydroxyl and carboxyl groups. This disruption of polymer–polymer interactions enhanced chain mobility, leading to increased flexibility and decreased stiffness in the films. Additionally, the higher solids content in the casting solutions of the anthocyanin-containing films contributed to greater thickness, which may have influenced stress distribution and deformation behavior under tensile load.

The differing pH conditions during film formation (pH 3 for FCA and pH 5 for FSA) could further modulate the anthocyanin structure, altering their interactions with the CMC matrix and partially explaining the observed differences in mechanical performance. These results are consistent with previous reports by Liu et al.^43^ and Liang et al.,^44^ who observed decreased maximum tensile strength and increased elongation in anthocyanin-incorporated films, confirming the plasticizing effect of these compounds.

### Fourier Transform Infrared Spectroscopy (FTIR)

The FTIR spectra of the films is presented in Figure 3. In the spectrum of the control film (FC), composed solely of CMC and glycerol, the absorption band observed between 1026 and 1107 cm^–1^ confirms the C–O stretching of the ether group present in the CMC structure.^45^

**Figure 3:**
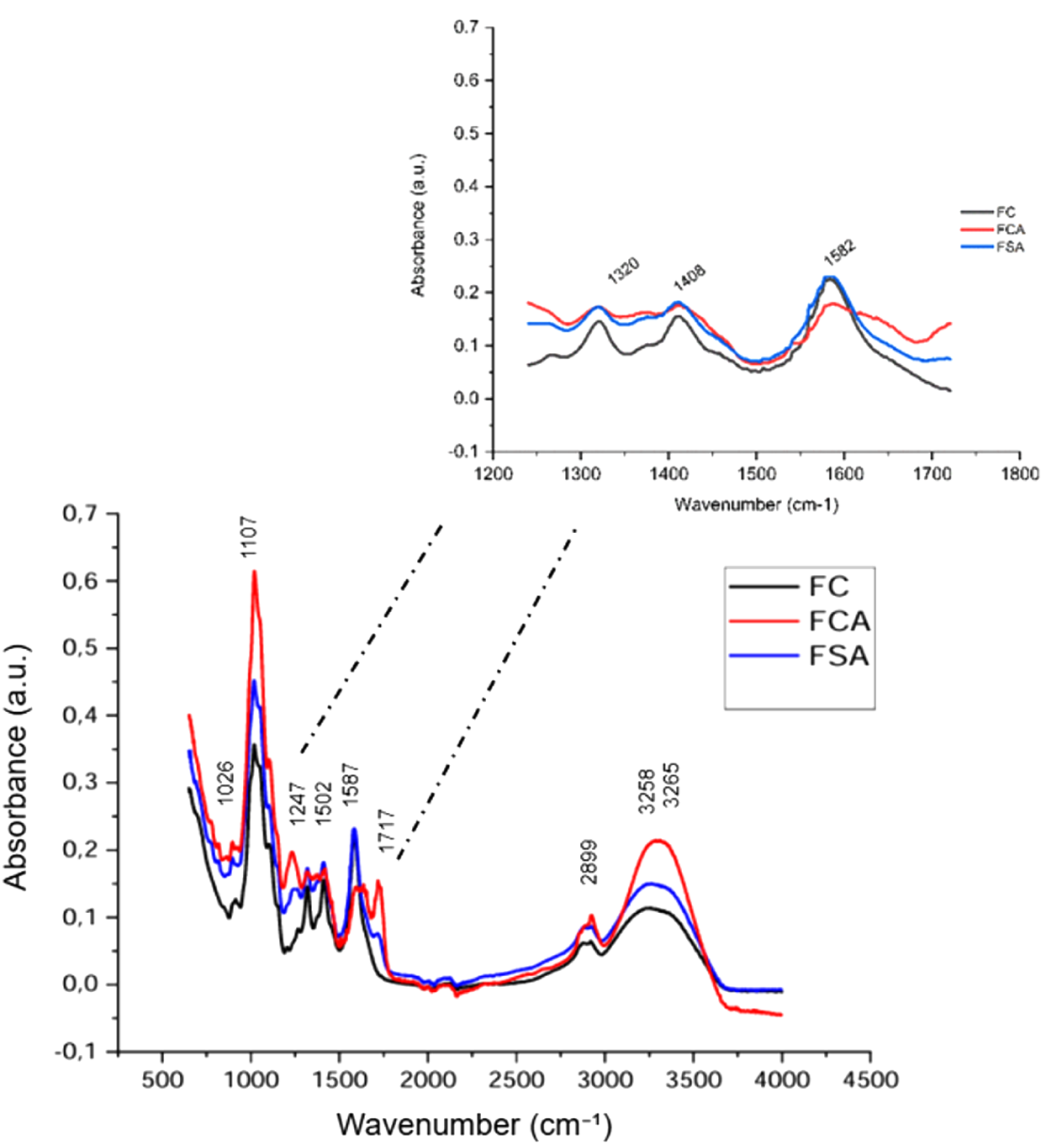
Infrared spectra of FC, FSA, and FCA films. The inset shows a magnified view in the 1240–1720 cm^–1^ region.

According to Li et al.,^46^ the peak at 1026 cm^–1^ can be attributed to the C–O–C stretching vibration, which is characteristic of this material. The peaks at 1502 and 1587 cm^–1^ correspond to the asymmetric and symmetric stretching vibrations of the carboxyl group in the polymer.

Similar results were reported by Tongdeesoontorn et al.,^47^ who identified peaks around 1592 cm^–1^ in CMC film spectra, attributed to the asymmetric vibration of the COO^–^ group. The peak at 2899 cm^–1^ was assigned to the stretching vibration of CH groups in the polymer, whereas the broad band observed at 3258 cm^–1^ is associated with O–H stretching of the CMC.^45^

The FTIR spectrum of the FSA film exhibited a similar pattern to that of the FC film, except peaks at 1247 and 1717 cm^–1^, which indicate the presence of phenolic and aldehydic groups, respectively, originating from the anthocyanins. These peaks confirm the incorporation of the extract into the polymer matrix. In addition, the broad band at 3258 cm^–1^ increased in intensity and shifted to 3265 cm^–1^. According to Roy et al.,^48^ the peak around 3265 cm^–1^ is associated with O–H stretching of anthocyanins, suggesting weaker polymer–polymer interactions and a greater presence of carboxylic groups derived from the anthocyanins.

The spectrum of the FCA film showed a distinct pattern compared to the FC film. The shifts and new peaks observed in FCA indicate that the lower pH promotes stronger interactions between anthocyanins and the CMC matrix, evidencing the impact of acidification on molecular interactions. This was evidenced by the appearance of new peaks at 859 cm^–1^, associated with aromatic compounds, and 1228 cm^–1^, attributed to phenolic groups, as well as a slight shift in the 1315 cm^–1^ peak related to C–H stretching, suggesting structural changes in the anthocyanins. According to Li et al.,^46^ the band at 1587 cm^–1^, observed in the FC film, intensified and shifted to 1720 cm^–1^, reflecting C = C stretching vibrations of the anthocyanin aromatic ring and its interaction with the polymer. Similar to FSA, the FCA film showed an increase in the band at 3258 cm^–1^, with a shift to 3297 cm^–1^, possibly due to the addition of HCl, which may have enhanced interactions between anthocyanins and the CMC matrix.

Seslija et al.^49^ reported that FTIR spectra of CMC films exhibit characteristic bands at 3340, 2920, and 1600 cm^–1^, corresponding to OH, C–H, and COO– stretching vibrations, respectively. Liu et al.,^50^ when analyzing CMC films containing grape pomace extract, identified a broad band in the 3200–3500 cm^–1^ region, attributed to −OH groups present in polyphenols, CMC, and anthocyanins. Additionally, axial deformation bands for C–H stretching between 3000 and 2800 cm^–1^ were associated with aliphatic chains, related to glycerol. In the 1500–1700 cm^–1^ range, symmetric axial deformation of the C=C bond in phenolic rings was also observed, which is consistent with the results obtained in the present study.

The FTIR spectra confirm that anthocyanins were successfully incorporated into the CMC matrix in the FSA films and that this interaction was intensified in the presence of HCl in the FCA films. Considering that the mechanical properties of the films are dependent on intermolecular interactions among their components,^48^ the FTIR data supports the results obtained in the mechanical property analyses.

### Solubility and Water Vapor Permeability

The FC film exhibited complete water solubility (100%) (Table 3), whereas the films containing anthocyanin extract showed a significant reduction in solubility. Film dissolution in aqueous media occurs through interactions between solute and solvent molecules. CMC is highly water-soluble due to the presence of hydrophilic groups, such as carboxyl (−COOH) moieties, which readily form hydrogen bonds with water molecules. Similarly, anthocyanins are also hydrophilic and water-soluble, owing to hydroxyl (−OH) groups in their structure that interact with water through hydrogen bonding.

**Table 3:** Aqueous Solubility and Water Vapor Permeability of FC, FCA, and FSA Films^a^

Film	Aqueous solubility (%)	WVP (g/m.s.mmHg)
FC	100 ± 0^A^	0.56 ± 0.17^A^
FCA	32.48 ± 7.45^B^	0.69 ± 0.53^A^
FSA	20.40 ± 2.79^C^	0.41 ± 0.04^A^

a Values followed by the same letter in the same column do not differ significantly (Tukey’s test, *P* < 0.05).

In the films containing anthocyanin extract, interactions between anthocyanin molecules and the CMC matrix may have reduced the availability of free hydrophilic groups to interact with water, resulting in decreased solubility. These interactions likely involve the carboxyl groups of CMC, which may become unavailable for hydrogen bonding with water, thereby rendering the films less soluble.

Among the films formulated with anthocyanins, it was observed that medium acidification affected solubility. The addition of HCl promoted stronger interactions between CMC and anthocyanins, as evidenced by the FTIR spectra. However, such interactions may not involve water molecules directly, which could limit full solubilization. Specifically, the FCA films exhibited greater solubility than the FSA films, possibly due to the acidic pH and the enhanced structural stability of anthocyanins under acidic conditions. These results confirm that the pH of the film-forming solution is an independent factor influencing solubility and polymer–anthocyanin interactions, facilitating the formation of less resistant intermolecular bonds and easier hydrolysis of the matrix.

Costa et al.^5^ explains that the incorporation of bioactive compounds, such as anthocyanins, can reduce the solubility of polymeric films due to the formation of interaction networks between the polymer and the incorporated compounds. These networks restrict chain mobility and reduce the number of free polar groups available for interaction with the solvent. Therefore, although anthocyanins may enhance the stability and functionality of the films, they may also compromise water solubility.

The films exhibited similar water vapor transmission rates, regardless of the incorporation of anthocyanins. Although FCA and FSA films showed greater thickness compared to the control film (FC), the presence of anthocyanins did not result in a significant reduction in WVP.

This behavior may be attributed to the restructuring of the polymer matrix due to interactions between anthocyanins and CMC, leading to the formation of a more compact network. However, this network was not sufficiently dense to prevent water vapor diffusion. The resulting microstructure may contain pores and channels that allow vapor passage, thereby explaining the comparable WVP values among the treatments.

These findings are consistent with Costa,^5^ who observed that films based on jackfruit starch with black grape anthocyanins exhibited increased thickness due to the addition of solids, without a corresponding decrease in water vapor permeability. In contrast, Hoffmann et al.^51^ reported a slight reduction in WVP for films containing jabuticaba anthocyanins, suggesting that the interaction between anthocyanins and the polymer matrix depends on the polymer source and the phenolic composition of the extract.

Therefore, the results suggest that the increased thickness of FCA and FSA films is more strongly associated with higher solid content than with polymer chain disruption or spacing. Consequently, the internal structure of the films was not sufficiently altered to provide an effective barrier against vapor diffusion, and the films showed similar moisture barrier performance, despite differences in thickness.

### Thermogravimetric Analysis (TGA) and Differential Thermal Analysis (DTA)

Thermogravimetric analysis (TGA) was performed to assess the thermal stability and decomposition events of the films, as shown in Figure 4. For the control film (FC), thermal degradation occurred in two main stages. The first stage was observed between 26.47 and 134 °C, with a mass loss of approximately 19%, attributed to the evaporation of free water present in the film matrix. This event reflects the removal of moisture not chemically bound to the polymer, characterizing the loss of surface or adsorbed water.

**Figure 4:**
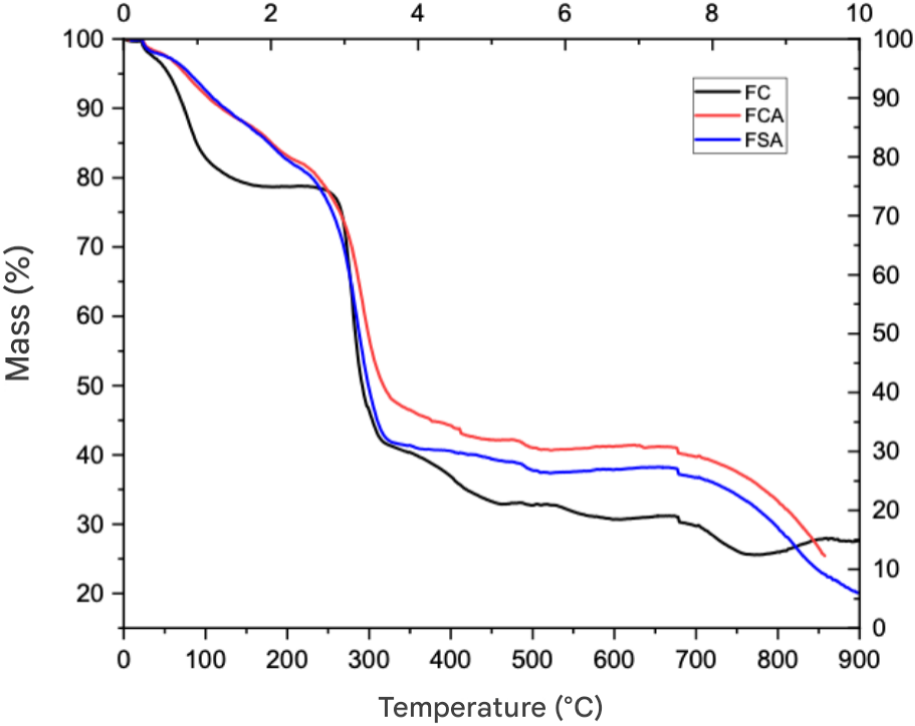
Thermogram of FC, FCA, and FSA films.

The second stage of mass loss occurred between 252 and 790 °C, with a mass reduction of around 50%, corresponding to the decomposition of the main CMC chain and the volatilization of the plasticizer (glycerol) present in the formulation. This behavior aligns with literature reports, as both the polymer and plasticizer exhibit high thermal decomposition points, leading to degradation at elevated temperatures.

In the films incorporated with anthocyanins (FSA and FCA), the initial mass loss began immediately after the melting process, indicating that the addition of the extract altered the thermal behavior and modified the structure of the polymer matrix. In the FCA film, mass loss was approximately 69%, while the FSA film exhibited a greater loss of around 74%. These events occurred within the temperature ranges of 652–894 °C, respectively.

Such variations may be attributed to enhanced interactions between anthocyanins and the polymer matrix, potentially influencing the thermal decomposition properties of the films. The greater mass loss observed in FSA and FCA suggests that the presence of anthocyanins and their intermolecular interactions with CMC contributed to more intense volatilization of the components during heating. Additionally, the presence of phenolic compounds may interfere with the thermal stability of the matrix, leading to shifts in the decomposition temperatures. However, these changes reflect modifications in the degradation pathway rather than premature thermal degradation of the films.

TGA data demonstrated that anthocyanin incorporation significantly affected the thermal stability of the films, as evidenced by the differences in degradation temperatures and mass loss rates between the control and anthocyanin-containing films. Despite these differences, the main decomposition events remained within temperature ranges compatible with conventional processing and application conditions for biodegradable packaging materials, indicating that the overall thermal stability of the films was preserved. These thermal differences are particularly relevant for applications where thermal resistance is critical, such as functional food packaging or biomaterials exposed to unstable thermal environments. Moreover, the observed changes in thermal behavior reflect potential alterations in the molecular interactions among the film components, which could also impact mechanical and barrier properties.

The results indicated that the FC film had a higher moisture content compared to anthocyanin-containing films, especially within the range of 300–700 °C. This behavior may be explained by a greater amount of water retained in the control film, resulting in more pronounced mass loss during the initial thermal event. This loss is primarily associated with the evaporation of free water within the film matrix, highlighting the importance of moisture control for the thermal stability of the material.

During the first thermal event, all films showed mass loss attributed to the evaporation of water molecules or other volatile compounds, as also reported by Ezati.^52^ This phenomenon is typical of films based on hygroscopic polymers, such as CMC, which retain water during production. The plasticizer glycerol volatilized during the second stage, around 280 °C, which is consistent with its relatively low boiling point.

Similar thermal events have been reported in studies involving anthocyanin-containing films. Hoffmann et al.^51^ observed comparable patterns in starch-based films with anthocyanins from jabuticaba peel. Likewise, Teixeira et al.^3^ and Hussain et al.^56^ reported compatible thermal degradation events, reinforcing the trend of decomposition and volatilization of components such as water and plasticizers.

The DTA curves of the FC, FCA, and FSA films are presented in Figure 5. For the FC film, an endothermic event was observed at 78.6 °C (−12.8 μV), corresponding to the evaporation of water present in the film, in agreement with the TGA data. Additionally, exothermic peaks were recorded around 396 °C (17.3 μV), indicative of the degradation of CMC and glycerol, followed by another endothermic variation at 556 °C, representing the final decomposition of the film.

**Figure 5:**
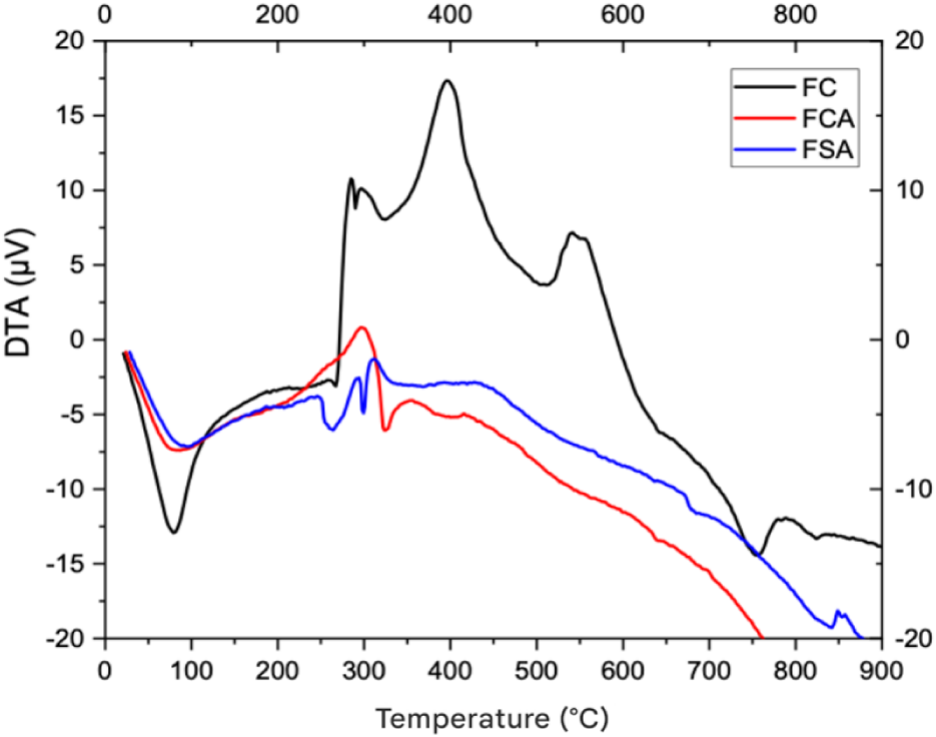
Thermal differential curves of FC, FCA, and FSA films.

In the FCA film, an endothermic event was observed around 294 °C (0.95 μV), associated with the melting of the material. After this point, the baseline showed a continuous endothermic trend, culminating in a decomposition event near 760 °C (−19.86 μV). The FSA film exhibited an endothermic event around 262 °C (−1.34 μV), attributed to the melting point. Two exothermic events were also recorded: one at 290 °C (−2.3 μV), possibly related to crystallization, and another at 311 °C (−1.15 μV), associated with the oxidation of the material.

The data indicate that the incorporation of anthocyanins did not significantly compromise the overall thermal stability of the films. This behavior is consistent with the findings of Cheng et al.^53^ who, through TGA analysis, reported similar mass loss patterns between starch-based films with and without red cabbage anthocyanins, with the first thermal event attributed to moisture loss around 30–105 °C.

In the DTA analysis, the film without anthocyanins exhibited exothermic peaks between 365 °C and 490 °C, while the anthocyanin-containing films showed thermal events between 240 °C and 540 °C. These results suggest that although the presence of anthocyanins slightly altered the thermal profile, it did not lead to substantial changes in the overall thermal characteristics of the films.

### Color Analysis of Films

The visual aspect of the films is presented in Figure 6. The incorporation of anthocyanin extract significantly modified the color properties of the films, as evidenced by the chromatic coordinates (L*, a*, b*, Δ*E*, and WI) presented in Table 4. The Δ*E* values indicated perceptible variations in hue, with the FCA and FSA films exhibiting distinct reddish colorations, differing in both intensity and saturation.

**Figure 6:**
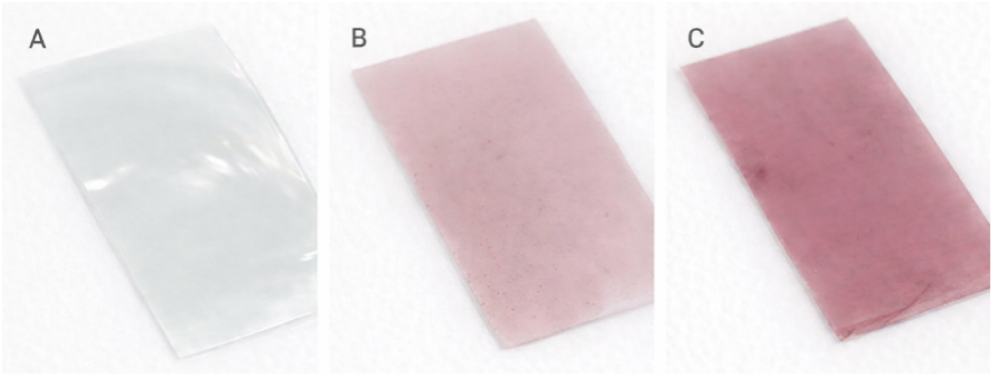
Visual appearance of FC (A), FSA (B), and FCA­(C) films.

**Table 4:**
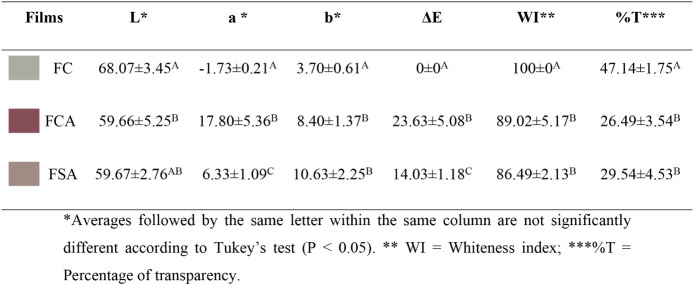
Color Coordinates, Whiteness Index, and Transparency Percentage of FC, FCA, and FSA Films

The acidification of the film-forming solution to pH 3, an intentional strategy to enhance the red coloration, was confirmed by the more intense hue observed in the films. This illustrates that acidification not only stabilizes anthocyanins in the flavylium cationic form but also modulates the functional and optical properties of the films independently from anthocyanin incorporation. This behavior is related to the chemical structure of anthocyanins, which are flavonoid compounds highly sensitive to pH. Under acidic conditions (pH 3), anthocyanins predominantly exist in their cationic form, responsible for the vivid red coloration.

The addition of HCl during film formulation promotes this condition, stabilizing the anthocyanins in their flavylium cationic form and resulting in deeper red tones. This form absorbs light in specific regions of the visible spectrum, thereby intensifying the red appearance of the films.^3,12^ These results indicate that the stabilization of anthocyanins in the flavylium cation form through acidification is an independent factor, capable of maintaining intense red coloration regardless of the total amount of anthocyanin incorporated in the film.

In the case of the FSA film (pH 5), although anthocyanins are present, the slightly higher pH favors the coexistence of both ionic and neutral molecular species, leading to a less intense coloration. At this pH range, anthocyanins tend to convert into carbinol pseudobase and chalcone forms, structural modifications that are nearly colorless or exhibit diminished pigmentation. This transformation accounts for the less saturated hue observed in the FSA film, in contrast to the FCA formulation, where the pH 3 condition preserves the cationic form and, consequently, the intense red color.

Beyond these chromatic alterations, the addition of anthocyanins also affected the whiteness index (WI) and the transparency of the films. The decrease in WI is associated with selective light absorption and reflection due to the presence of natural pigments, which impart color and reduce whiteness. Meanwhile, the reduction in transparency results from anthocyanin absorption in the blue and green regions of the visible spectrum, leading to increased opacity. These effects can be strategically utilized in specific applications, such as packaging for photosensitive foods or pH-indicating systems, where color intensity and variation serve as functional visual indicators.

The colorimetric attributes (L*, a*, b*, Δ*E*, and WI) of the FCA and FSA films were also evaluated as a function of pH (ranging from 3 to 10), as shown in Tables 5 and 6, respectively. In general, the chromatic characteristics of the films did not vary significantly with changes in the pH of the medium, except for the FCA film at pH 10, where the b* coordinate indicated a shift toward a yellowish hue. This behavior is consistent with the findings reported by Zhang et al.,^11^ who observed that under neutral or alkaline conditions, anthocyanins undergo structural transformations into *cis*-chalcone, which is associated with yellowish coloration.

**Table 5:**
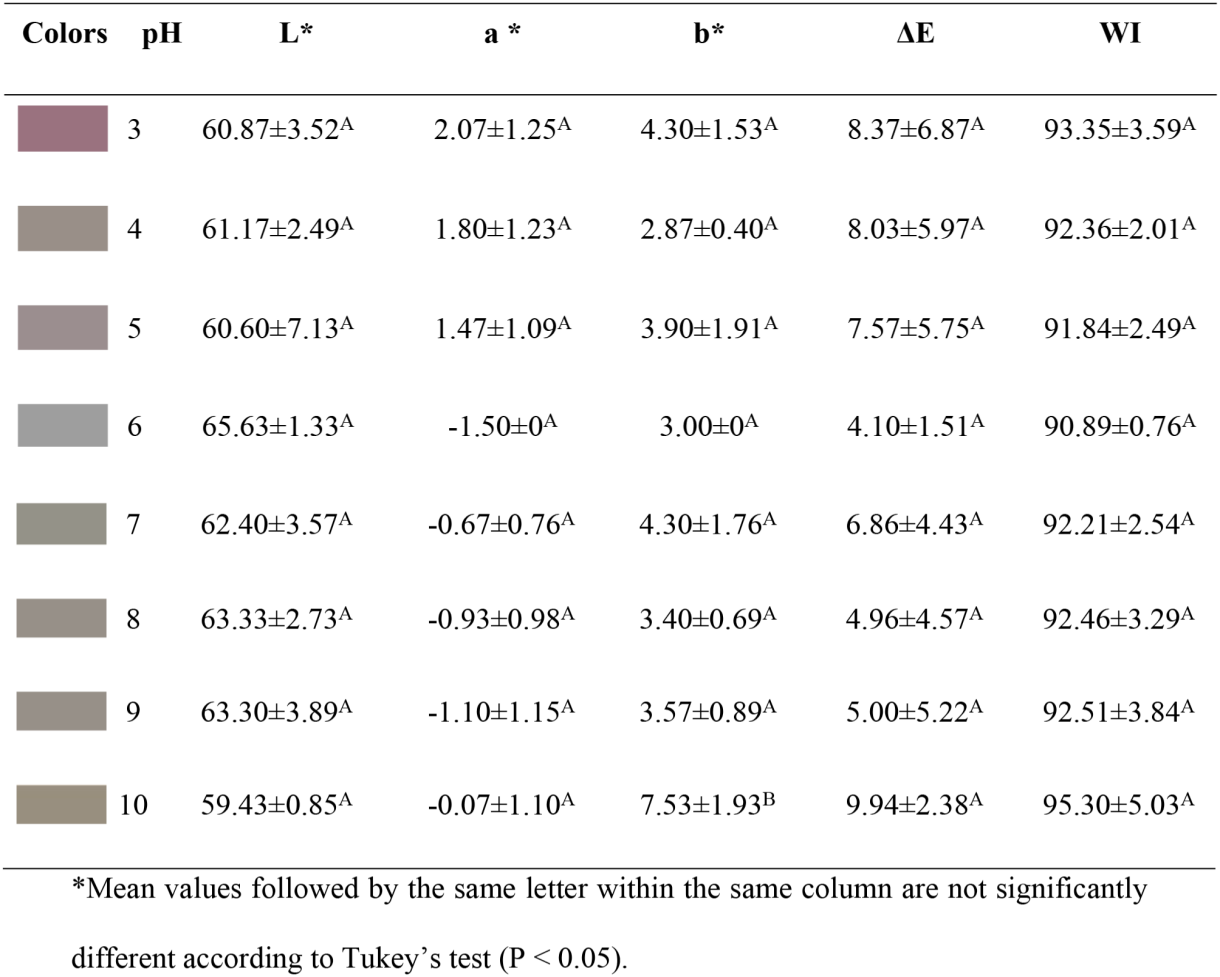
Color Coordinates and Whiteness Index of FCA Films at Different pH Values

**Table 6:**
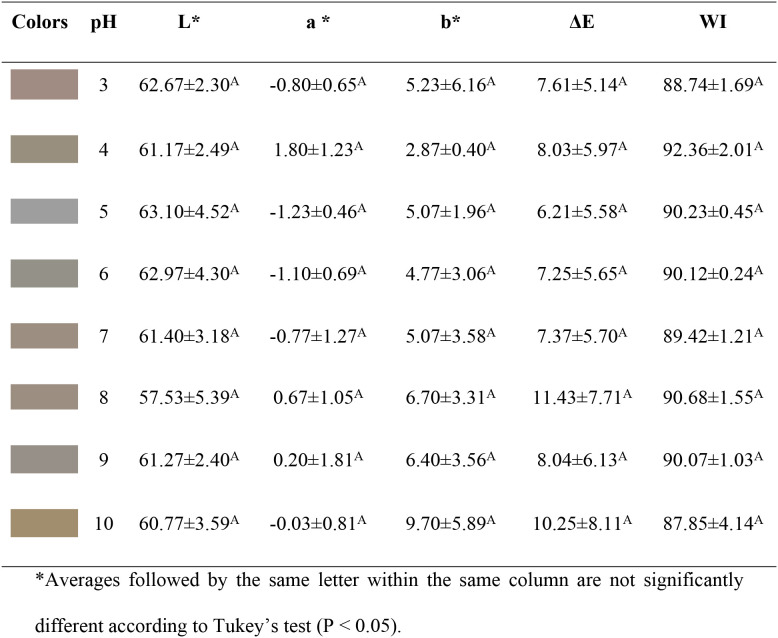
Color Coordinates and Whiteness Index of FSA Films as a Function of pH Variation in the Surrounding Medium

This limited chromatic variation across most of the evaluated pH range indicates a restricted pH responsiveness of the films, which can be attributed to strong polymer–anthocyanin interactions that stabilize the pigments within the CMC matrix and reduce their ability to undergo rapid structural transitions in response to external pH changes.

According to Yildirim-Yalcin et al.,^25^ the use of CMC provides good transparency to the films and is compatible with natural pigments. This good transparency is attributed to the homogeneous, amorphous structure of CMC, which allows light to pass through with minimal scattering. Despite this compatibility, the coloration observed after immersion of the films in buffer solutions (pH 3 to 10) exhibited low intensity, as shown in Figure 7, which may have influenced the colorimetric response observed.

**Figure 7:**
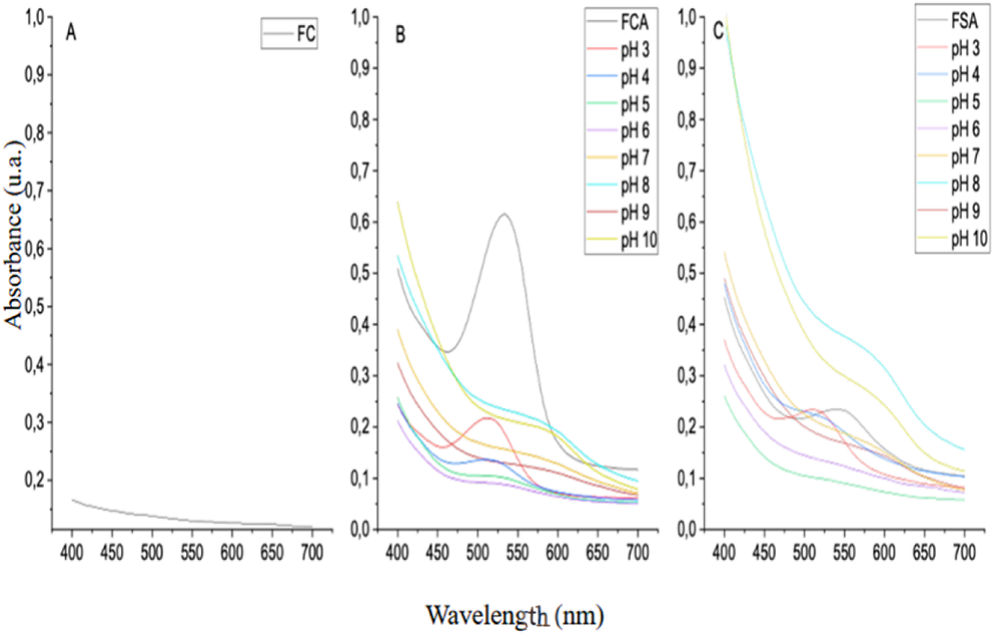
Absorption spectrum in the UV/Visible regions (400–700 nm) of the FC, FCA, and FSA films. (A) FC film; (B) FCA; and (C) FSA, at pH ranging from 3 to 10.

In a study conducted by Hoffmann et al.^51^ using starch-based films containing anthocyanins from jabuticaba, a maximum absorption peak in the UV–vis spectrum was observed at approximately 516 nm (pH 3). The absorbance decreased with increasing pH, indicating a hypochromic effect. This reduction is associated with anthocyanin deprotonation and the consequent alteration of their chemical structure. Moreover, no absorption peaks were observed in the pH range of 5–11, during which the solutions became colorless or slightly yellow.

Anthocyanins extracted from jabuticaba peels can exist in various structural forms depending on the pH. At pH values above 2, an equilibrium is established between the cationic form and the carbinol pseudobase, with the latter predominating around pH 6, resulting in a colorless appearance. Under alkaline conditions (pH > 7), deprotonation leads to the formation of chalcones, which are typically yellow, earthy, or brown.^54,55^

Figure 7 shows the absorption spectra (400–700 nm) of FC, FCA, and FSA films. The FC film did not exhibit any relevant absorption bands, regardless of pH. In contrast, the FCA film displayed peaks at 532 nm (pH 3), 511 nm (pH 4), and 589 nm (pH 8 and 10). No characteristic absorption bands were observed at pH values 5 to 7 and 9. The FSA film exhibited a similar behavior, initially showing maximum peaks at 544 and 517 nm (pH 3), 515 nm (pH 4), and 570 nm (pH 8 and 10). No absorption bands were identified between pH 5 and 7 or at pH 9.

The results reinforce that the interactions between anthocyanins and polymers occur predominantly through hydrogen bonding, which is essential for the formation and stability of the films. This phenomenon highlights the influence of the specific chemical properties of anthocyanins and the relevance of molecular interactions in determining the structural and functional characteristics of the developed materials.

### Film Stability

The color coordinate data obtained in the first and eighth weeks of the films’ shelf life are presented in Table 7. The FCA and FSA films showed statistically significant differences compared to the FC film for all colorimetric attributes, as previously discussed. When compared to each other, the films with anthocyanin addition maintained significant differences in the a* coordinate after 8 weeks of storage. Furthermore, a significant reduction in the b* coordinate was observed, indicating a loss of coloration, with a shift toward more yellowish tones.

**Table 7:**
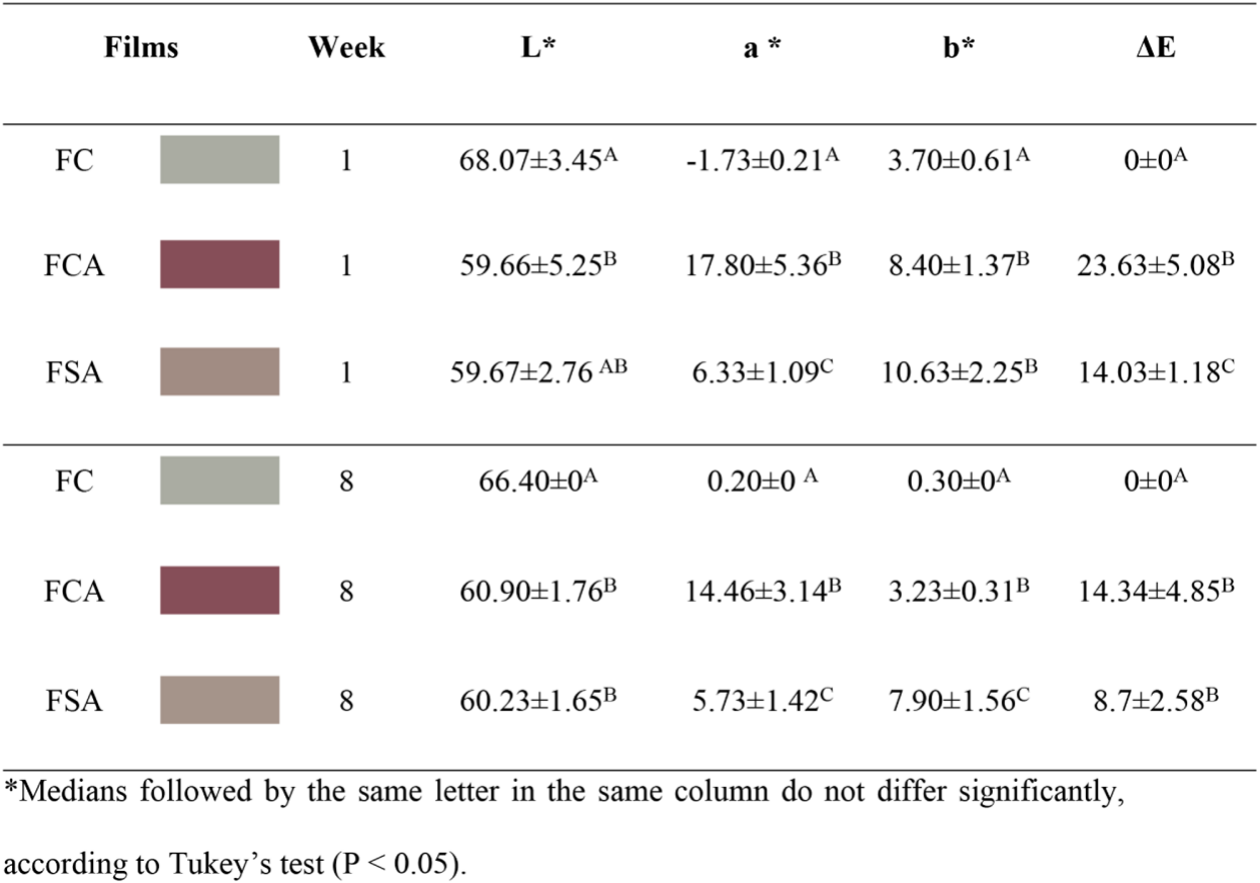
Color Coordinates of FC, FCA, and FSA Films After 8 Weeks of Storage

The color coordinates of FC and FCA films showed no statistical differences between the first and 8 weeks, indicating satisfactory chromatic stability during the storage period. On the other hand, the FSA film exhibited significant changes in the b* coordinate, resulting in a change in the Δ*E* value. These observations further highlight that acidification in FCA enhances color stability during storage compared to nonacidified films (FSA), confirming the protective role of low pH. However, visually, the color differences between the first and eighth weeks were not perceptible to the naked eye.

The statistical test demonstrated that the FC and FCA films maintained color stability for more than 50 days under the adopted storage conditions, highlighting the efficiency of the polymer matrix in preserving the optical properties of the incorporated natural pigments. These results are superior to those reported by Jiang et al.,^19^ who developed CMC and starch-based films incorporated with purple sweet potato anthocyanins and observed color stability for only 20 days of storage. Future studies should further evaluate the durability of color under different storage conditions, such as variable temperature, humidity, and light exposure, to better understand the practical applicability of these films.

### Application of the Films in a Dairy Matrix

The practical application of the FC, FCA, and FSA films was evaluated in pasteurized milk stored for up to 48 h, aiming to assess changes in the films color coordinates in response to pH variations, simulating spoilage conditions. Milk was selected as a model liquid food due to its common spoilage-related pH changes, providing a relevant system to evaluate the potential of pH-sensitive films.

Table 8 presents the L*, a*, and b* values of the films at 0, 24, and 48 h. In general, no statistically significant differences (*p* > 0.05) were observed in the films color coordinates over time, except for the L* value of the FSA film, which increased significantly after 48 h, indicating a slight change in brightness.

**Table 8:** Color Coordinate Values of the Films Applied to Pasteurized Milk

Samples	*L* ^a^	*a* ^a^	*b* ^a^	Δ*E*
**0 h**				
Control	70.17 ± 0.85^A^	-2.77 ± 0.85^A^	11.30 ± 2.03^A^	0.00 ± 0.00^A^
FC	68.07 ± 0.35^A^	-3.33 ± 0.38^A^	11.80 ± 2.01^A^	2.79 ± 0.24^A^
FCA	68.37 ± -1.56^A^	-1.13 ± 1.64^A^	8.90 ± 2.49^A^	3.67 ± 2.93^A^
FSA	67.8 ± 1.82^A^	-2.80 ± 1.47^A^	11.03 ± 3.09^A^	3.85 ± 1.13^A^
**24 h**				
Control	69.60 ± 0.95^A^	-4.17 ± 0.93^A^	15.83 ± 0.32^A^	1.07 ± 0.39^A^
FC	68.03 ± 1.16^A^	-5.07 ± 1.08^A^	16.13 ± 4.31^A^	4.04 ± 1.30^A^
FCA	68.17 ± 1.42^A^	-2.93±0.91^A^	12.87±3.47^A^	3.96 ± 3.16^A^
FSA	67.23 ± 1.19^A^	-3.57 ± 0.50^A^	16.33 ± 1.93^A^	2.88 ± 1.52^A^
**48 h**				
Control	64.83 ± 1.22^A^	-4.20 ± 0.62^A^	14.13 ± 0.64^A^	1.16 ± 0.37^A^
FC	65.67 ± 0.91^A^	-3.83 ± 0.50^A^	13.83 ± 2.14^A^	1.86 ± 0.47^A^
FCA	62.83 ± 2.72^A^	-2.80 ± 1.84^A^	14.33 ± 2.04^A^	3.26 ± 1.41^A^
FSA	67.47 ± 0.72^B^	-3.53 ± 1.48^A^	14.40 ± 4.69^A^	3.81 ± 1.82^A^

a Means followed by the same letter within the same column do not differ significantly according to Tukey’s test (*P* < 0.05).

To complement the individual color coordinate analysis, the total color difference (ΔE*) was calculated to provide an integrated evaluation of the perceptible color changes of the films over storage time. As shown in Table 8, ΔE* values did not differ significantly among the films at 0, 24, and 48 h (*p* > 0.05), remaining below values typically associated with easily perceptible visual changes. This result corroborates the absence of evident chromatic variation observed during milk storage and confirms that the minor fluctuations in L*, a*, and b*, even under a decrease in milk pH from 7.0 to 6.0, were not sufficient to generate a noticeable overall color change or to indicate spoilage.

Notably, the FCA film, prepared at pH 3, retained its red hue due to stabilization of anthocyanins in the flavylium cation form, while the FSA film, prepared at pH 5, contained anthocyanins partially in the colorless carbinol form. Although these pH-dependent differences affected initial color intensity, the liquid milk matrix minimized observable changes due to dilution and partial leaching of anthocyanins. Therefore, the limited color response observed in milk is mainly associated with the interaction between the liquid matrix and the film structure, rather than being solely attributed to anthocyanin leaching. This behavior indicates that, under mildly acidic conditions typical of early milk spoilage, the colorimetric response of the films is constrained by the liquid nature of the matrix rather than by the intrinsic pH sensitivity of the anthocyanins.

This limitation can be primarily attributed to the dilution effect and restricted film–matrix interaction in the liquid system, coupled with the inherently reduced sensitivity of anthocyanins under mildly acidic conditions, as previously reported by Hoffmann et al.^51^ To improve the practical applicability of these films as intelligent indicators, future research should focus on strategies such as encapsulating anthocyanins to minimize leaching or evaluating their performance in solid and semisolid food matrices, where greater contact and retention of bioactive compounds could enhance colorimetric responsiveness.

These results suggest that, under the tested conditions, the developed films did not exhibit sufficient colorimetric sensitivity to function as spoilage sensors in a liquid matrix. These findings contrast with the study by Hoffmann et al.,^51^ who observed perceptible color changes in starch-based films incorporated with jabuticaba anthocyanins when applied to milk undergoing spoilage.

The discrepancy may be associated with the matrix composition, polymer–pigment interactions, and the sensitivity limits of the films developed in this study. Moreover, the liquid nature of the matrix may have favored the dispersion of phenolic compounds and attenuated the chromatic response at moderately acidic pH, limiting the visibility of changes.

These results highlight the complexity of the interaction between intelligent films and liquid food matrices, evidencing that different physicochemical characteristics of the food can modulate the response. Therefore, the application of the films in solid or semisolid matrices, which favor greater contact and retention of active compounds, represents a promising opportunity to enhance their functionality as visual indicators.

## Conclusion

The results obtained demonstrated that the production of the films is feasible and that they possess suitable structural and functional properties for applications in biodegradable packaging. The incorporation of anthocyanin extract conferred sensitivity to pH variations, responding distinctly to different environmental conditions, which reinforces their potential as visual indicators in intelligent systems. Specifically, the FCA film showed the highest red coloration (*a* = 17.80 ± 5.36; Δ*E* = 23.63 ± 5.08), while the FSA film exhibited moderate red coloration (*a* = 6.33 ± 1.09; Δ*E* = 14.03 ± 1.18). Acidification at pH 3 also enhanced chromatic stability, as FCA maintained a* = 14.46 ± 3.14 after 8 weeks, compared to a* = 5.73 ± 1.42 for FSA.

Acidification at pH 3 enhanced red coloration and improved chromatic stability during storage, while anthocyanin incorporation reduced solubility without affecting thermal stability. Despite these positive effects, the films showed limited colorimetric sensitivity when applied in liquid milk under the evaluated conditions, which is mainly associated with the characteristics of the liquid matrix, suggesting that their potential use is greater in solid or semisolid foods.

Future studies should explore the performance of these films in diverse food matrices, across broader pH ranges and under different storage conditions, and further optimize film formulations to maximize color intensity, stability, and pH responsiveness.

## Data Availability

All data supporting the findings of this study are included in the manuscript.
